# Family-based Bayesian collapsing method for rare-variant association study

**DOI:** 10.1186/1753-6561-8-S1-S37

**Published:** 2014-06-17

**Authors:** Liang He, Janne M Pitkäniemi

**Affiliations:** 1 University of Helsinki, Hjelt Institute, Department of Public Health, PO Box 41, FI-00014 Helsinki, Finland; 2 Finnish Cancer Finnish Cancer Registry, Institute for Statistical and Epidemiological Cancer Research, Pieni Roobertinkatu 9, FI-00130 Helsinki, Finland

## Abstract

In this study, we analyze the Genetic Analysis Workshop 18 data to identify the genes and underlying single-nucleotide polymorphisms on 11 chromosomes that exhibit significant association with systolic blood pressure. We propose a novel family-based method for rare-variant association detection based on the hierarchical Bayesian framework. The method controls spurious associations caused by population stratification, and improves the statistical power to detect not only individual rare variants, but also genes with either continuous or binary outcomes. Our method utilizes nuclear family information, and takes into account the effects of all single-nucleotide polymorphisms in a gene, using a hierarchical model. When we apply this method to the genome-wide Genetic Analysis Workshop 18 data, several genes and single-nucleotide polymorphisms are identified as potentially related to systolic blood pressure.

## Background

Current studies suggest that a large number of common variants identified in genome-wide association studies (GWAS) as being associated with various complex diseases can account for only a small portion of phenotype variation [1]. With the advent of next-generation sequencing, attention has focused on rare variants (RVs), such as single-nucleotide polymorphisms (SNPs) with a minor allele frequency (MAF) of less than 1%. Traditional single-marker methods lose statistical power for detecting RV association because of their rare occurrence. In the last few years, however, a variety of methods have been developed, including the combined multivariate and collapsing (CMC) method [2] and the weighted sum (WS) method [3]. More sophisticated methods that are robust to different variant effects include the kernel-based adaptive cluster (KBAC) method [4], the C-alpha test [5], and the sequence kernel association test (SKAT) [6].

These methods, however, all assume that individuals are independently sampled and are, therefore, vulnerable to the influence of population stratification. Exploring marker transmission within a family avoids the issues of population stratification. More important, once anRV enters a family, it can segregate to other family members so that copies of the minor allele are enriched in the data. This could potentially increase the statistical power of family-based approaches.

Here we propose a novel family-based Bayesian collapsing model (FBCM) capable of identifying associations of RVs and genes with quantitative phenotypes. The method builds on the hierarchical quantitative transmission disequilibrium test (HQTDT) [7]. Compared to classical statistical methods, the Bayesian framework incorporates prior information, thereby providing an alternative approach to situations in which factors affecting the power of the test, such as the MAF of the SNP, play an important role [8]. We combine HQTDT with the idea of collapsing under a Bayesian framework. Then we expand the model in a data-driven manner by utilizing random effects to model the signals of individual rare variants within a gene.

## Methods

### Family-based Bayesian collapsing model

Several statistical models based on the Bayesian framework, such as model selection [9] and multiple regression [10], have been proposed for RV association detection. Because of the large scale of model space and matrix calculation, these approaches suffer from impractical computational time if a full joint posterior distribution is required. Although most Bayesian methods endeavor to employ various optimization or approximation algorithms to obtain a point estimate, the loss of uncertainty information on the estimate means the significance of the estimate cannot be evaluated. In this paper, we propose a Bayesian model that aims to efficiently generate a full posterior distribution without the loss of model space, and is viable for family-based genome-wide association analysis. The central idea comes from collapsing RVs and modeling their effects using variant-specific random effects. In some cases, it is probable that an RV is enriched in certain pedigrees while being very rare in others. Thus, among a group of RVs, some can be both neutral and associated with the phenotype through population stratification. To solve this problem, the RVs are collapsed in 2 orthogonal components to adjust for the possible population stratification.

Consider a candidate gene that contains *Κ *diallelic loci (in this paper, a locus always refers to the location of a SNP) with MAF less than 1%. Given a set of *i*= 1,..., *M *nuclear families, each of which contains *n_i _*siblings so that the total number of offspring is $\overset{M}{\underset{i=1}{\sum }}n_{i}=N,$ we define the coded genotypic score *G_ijk _*for the *j*^th ^child in the *i*^th ^family as the number of minor alleles at the *k*^th ^locus. It is assumed that both parents of each child are available, and, correspondingly, the genotypic scores of the parents at the *k*^th ^locus in the *i*^th ^family are denoted by *GM_ik _*and *GF_ik_*, respectively, for the mother and father. Conditional on the parental genotypes, the expected score for the offspring in the *i*^th ^family at the *k*^th ^locus under mendelian law is $GE_{ik}=\frac{GM_{ik}+GF_{ik}}{2}$. Furthermore, the deviation of the genotypic score for the *j*^th ^child in the *i*^th ^family at the *k*^th ^locus, which is denoted by *D_ijk_*, is *G_ijk_*− *GE_ik_*. For technical reasons, we add a pseudolocus *k *= 0 and define *GE*_*i0 *_= *D*_*ij0 *_= 0. When at least 1of the parents carries the copies of minor alleles at a locus, it is then possible to observe deviation in offspring at this locus. However, given a moderate set of variants, it is very unlikely for an individual to harbor minor alleles at more than 2 causal variants. For instance, when MAF is 0.005 and there are 50 independent causal RVs, the probability of an individual having minor alleles at more than 2 loci is $\text{1}-0.\text{9}{\text{9}}^{\text{5}0}-\text{5}0\;\cdot \;0.\text{9}{\text{9}}^{\text{49}}\cdot \;0.0\text{1}-\frac{50.49}{2}\cdot \;0.\text{9}{\text{9}}^{\text{48}}\cdot \;0.0{\text{1}}^{\text{2}}\approx \text{1}.\text{38}\%$. Thus, by taking advantage of the rare occurrence of copies of minor alleles, for each individual we consider at most 2 loci that have nonzero deviation in a candidate gene. These are indexed by *r_ij _*and *s_ij_*, which are defined below.

$$r_{ij}=\left\{\begin{array}{cc} k, & if\;individual\;j\;in\;family\;i\;has\;deviation\;at\;least\;at\;1\;locus\\ 0, & and\;the\;locus\;with\;the\;smallest\;MAF\;is\;indexed\;by\;k,\;otherwise. \end{array}\right)$$

$$s_{ij}=\left\{\begin{array}{cc} k, & if\;individual\;j\;in\;family\;i\;has\;deviation\;at\;more\;than\;1\;locus\\ 0, & and\;the\;locus\;with\;the\;second\;smallest\;MAF\;is\;indexed\;by\;k,\;otherwise. \end{array}\right)$$

This method dramatically shortens computational time by avoiding large-scale matrix computation in Gibbs sampling. If an individual has nonzero deviation at fewer than 2 loci, both or *s_ij _*are 0. Those with the smallest MAFs are selected if an individual has more than 2 loci with nonzero deviation. Thus, more emphasis is placed on those with smaller MAFs because deleterious functional variants tend to have low frequencies [12]. Given that RVs often do not exhibit strong linkage disequilibrium (LD) with either rare or common SNPs [11], for a moderate number of RVs such approximation loses much less information than do naive collapsing methods. Moreover, including 2 loci enables the model to detect the additive effect combination of 2 RVs.

Let *y_ij _*denote the quantitative phenotype for the *j*^th ^child in the *i*^th ^family. The relationship between the phenotype and the set of RVs in the candidate gene can be expressed by a hierarchical model

(1) $$y_{ij}=\mu +\beta_{1}.\left(\alpha_{r_{ij}}.\;D_{ijr_{ij}}+\alpha_{s_{ij}}.\;D_{ijs_{ij}}\right)+\beta_{2}.\left(\gamma_{r_{ij}}.GE_{ir_{ij}}+\gamma_{s_{ij}}.GE_{is_{ij}}\right)+\varphi_{i}+\epsilon_{ij}$$

$$\phi_{i}\sim N\left(0,\sigma_{\phi }^{2}\right)$$

$$\epsilon_{ij}\sim N\left(0,\sigma_{\epsilon }^{2}\right)$$

$$r_{ij},s_{ij}\;\epsilon \;0,1,\ldots ,K$$

where *µ *is the global intercept and *ε_ij _*is the random error. The genotypic score is decomposed into within-family and between-family components, and the construction of formula (1) guarantees the orthogonality of those 2 components. Inference based on *β*_1 _provides a stratification-resistant within-family test, while *β*_2 _estimates the genetic effect resulting from stratification. As a result of the limitation of the sample size for the inference and the fact that the variance components are not our major interest in this study, the family-level variable *φ_i _*is modeled as a random effect. This enables us to capture the between-family variance that includes the influence of the family-specific environmental factor.

The vectors of variant-specific random effects $\tilde{\alpha }=\left(\alpha_{0},\alpha_{1},\cdots \;,\alpha_{\text{k}}\right)$ and $\tilde{\gamma }=\left(\gamma_{0},\gamma_{1},\cdots \;,\gamma_{\text{k}}\right)$ modulate the within-family and between-family global effects *β*_1 _and *β*_2_, respectively. *r_ij _*and *s_ij _*are individual-specific indices of which elements in $\tilde{\alpha }$ and $\tilde{\gamma }$ contribute to the *j*^th ^child in the *i*^th ^family. It is possible that some of the RVs are neutral, but may be associated with the phenotype through population stratification. Ignoring this possibility will not only inflate the type I error rate, but also will introduce noise after collapsing. By modeling these 2 situations using $\tilde{\alpha }$ and $\tilde{\gamma }$ separately, formula (1) (below) manages to detect the association, accounting for neutral RVs as well as population stratification.

Although it is less common to observe LD between RVs compared to common variants, only independent representative SNPs are selected and included in the analysis. Because 2 loci at most are taken into account for each individual, such selection improves the accuracy and efficiency of the model.

### Prior distributions for random effects

The multiplicative relation in the 2 pairs (i.e., *β*_1 _and $\tilde{\alpha }$, *β*_2 _and $\tilde{\gamma }$) may result in a nonidentifiable model. To ensure identifiability, $\tilde{\alpha }$ and $\tilde{\gamma }$ --except for $\alpha_{0}$ and $\gamma_{0}$, which are random effects of the pseudolocus and sampled from *Bern*(0)--are selected to be independently sampled from Bernoulli distributions with hyper-parameters *p_k _*and *q_k_. β*_1 _and *β*_2 _are given a noninformative normal prior distribution with some variance $\sigma_{\beta }^{2}$, that is,

(2) $$\begin{array}{c} \alpha_{k}\sim Bern\left(p_{k}\right),\;p_{k}\sim Beta\left(1,1\right)\\ \gamma_{k}\sim Bern\left(q_{k}\right),\;q_{k}\sim Beta\left(1,1\right)\\ \beta_{1}\sim N\left(0,\alpha_{\beta }^{2}\right),\;\beta_{2}\sim N\left(0,\alpha_{\beta }^{2}\right) \end{array}$$

The *k*^th ^variant is treated as associated when *α_k _*= 1; otherwise it is neutral. The hyperparameter *p_k _*is the predictor for *α_k _*and can be regarded as the probability of the *k*^th ^variant being associated. With such a prior distribution for *α_k_*, the model actually selects the optimal group of associated RVs in a data-driven way and then collapses them together.

### Bayesian inference

To investigate the gene-level association, we wish to test the hypothesis *β*_1 _= 0. However, in a Bayesian framework, this hypothesis cannot be evaluated directly because the posterior distribution of *β*_1 _is continuous. Instead, we can conduct a composite hypothesis test:

$$H_{0}:\left|\beta_{1}\right|\;\leq \;\epsilon \;\;or\;\sum_{k-1}^{K}\alpha_{k}=0$$

(3) $$H_{1}:\left|\beta_{1}\right|>\;\;\epsilon \;\;and\;\overset{K}{\underset{k=1}{\sum }}\alpha_{k}>0,$$

Where ∈ is a small positive number. Although in principle the choice of ∈ is arbitrary, a too small ∈ might inflate the estimate error resulting from the numerical approximation. So we set ∈ as $0.\text{2}*{\hat{\sigma }}_{\epsilon },$ where ${\hat{\sigma }}_{\epsilon }$ is the estimated standard deviation for random error. The Bayes factor (BF) is a good way to summarize the evidence provided by the data in favor of one statistical model over another while also taking into account the complexity of a model. Note that the BF can be expressed using the ratio of the odds of the posterior distribution, which can be obtained approximately by the Monte Carlo Markov chain (MCMC) method, to the prior odds. For the prior distribution described above, the prior odds are calculated as

$\frac{P\left(\left|\beta_{1}\right|>\epsilon \cap \sum_{k=1}^{K}\alpha_{k}>0\right)}{P\left(\left|\beta_{1}\right|\leq \epsilon \cup \sum_{k=1}^{K}\alpha_{k}=0\right)}=\frac{\left(1-erf\left(\frac{\epsilon }{\sqrt{2}\sigma_{\beta }}\right)\right)\left(1-0.5^{K}\right)}{erf\left(\frac{\epsilon }{\sqrt{2}\sigma_{\beta }}\right)\left(1-0.5^{K}\right)+0.5^{K}},$ (4)

where *erf *(•) is the error function, defined as: $erf\left(x\right)=\frac{2}{\sqrt{\pi }}\int_{0}^{x}e^{-t^{2}}dt$. Thus, the hybrid BF can be obtained by

$$BF\left(H_{1}:H_{0}\right)=\frac{\hat{P}\text{(}\left|\beta_{1}\right|>\epsilon \cap \sum_{k=1}^{K}\alpha_{k}>0|Data\text{)}}{\hat{P}\text{(}\left|\beta_{1}\right|\leq \epsilon \cup \sum_{k=1}^{K}\alpha_{k}=0|Data\text{)}}/\frac{P\text{(}\left|\beta_{1}\right|>\epsilon \cap \sum_{k=1}^{K}\alpha_{k}>0\text{)}}{P\text{(}\left|\beta_{1}\right|\leq \epsilon \cup \sum_{k=1}^{K}\alpha_{k}=0\text{)}}$$

(5) $$BF\left(H_{1}:H_{0}\right)=\frac{\hat{P}\left(\left|\beta_{1}\right|>\epsilon \text{|}Data\right)}{\hat{P}\left(\left|\beta_{1}\right|\leq \epsilon \text{|}Data\right)}/\frac{P\left(\left|\beta_{1}\right|>\epsilon \right)}{P\left(\left|\beta_{1}\right|\leq \epsilon \right)},$$

where $\hat{P}\left(\left|\beta_{1}\right|>\epsilon \cap \overset{K}{\underset{k=1}{\sum }}\alpha_{k}>0|Data\right)$ and $\hat{P}\left(\left|\beta_{1}\right|\leq \epsilon \cup \overset{K}{\underset{k=1}{\sum }}\alpha_{k}=0|Data\right)$ are estimated from the posterior distribution approximated by the outputs of the MCMC method. If the BF exceeds a certain threshold, which is selected through simulations, we conclude that $\beta_{1}$ is significant. Once there is evidence of a global association, we can further assess the underlying RVs by investigating the marginal posterior distribution for α*_k, _k *ϵ 1,..., *Κ*. Note that if we treat α*_k _*as a model indicator, one way to quantify and summarize the posterior probabilities is to calculate the marginal BF, which is the ratio of the posterior odds to the prior odds of the same variable, defined as:

(6) $$BF\left(M_{1}\left(\alpha_{k}\neq 0\right):M_{0}\left(\alpha_{k}=0\right)\right)=\frac{\hat{P}\left(\alpha_{k}\neq 0|y\right)/P\left(\alpha_{k}\neq 0\right)}{\hat{P}\left(\alpha_{k}=0|y\right)/P\left(\alpha_{k}=0\right)},$$

The model is implemented using WinBUGS with 50,000 iterations, and the convergence is checked by investigating the autocorrelations for all parameters. We also simulate several chains with different initial values simultaneously, and evaluate convergence with the Gelman-Rubin convergence diagnostic tool [13].

## Results

**Figure 1:**
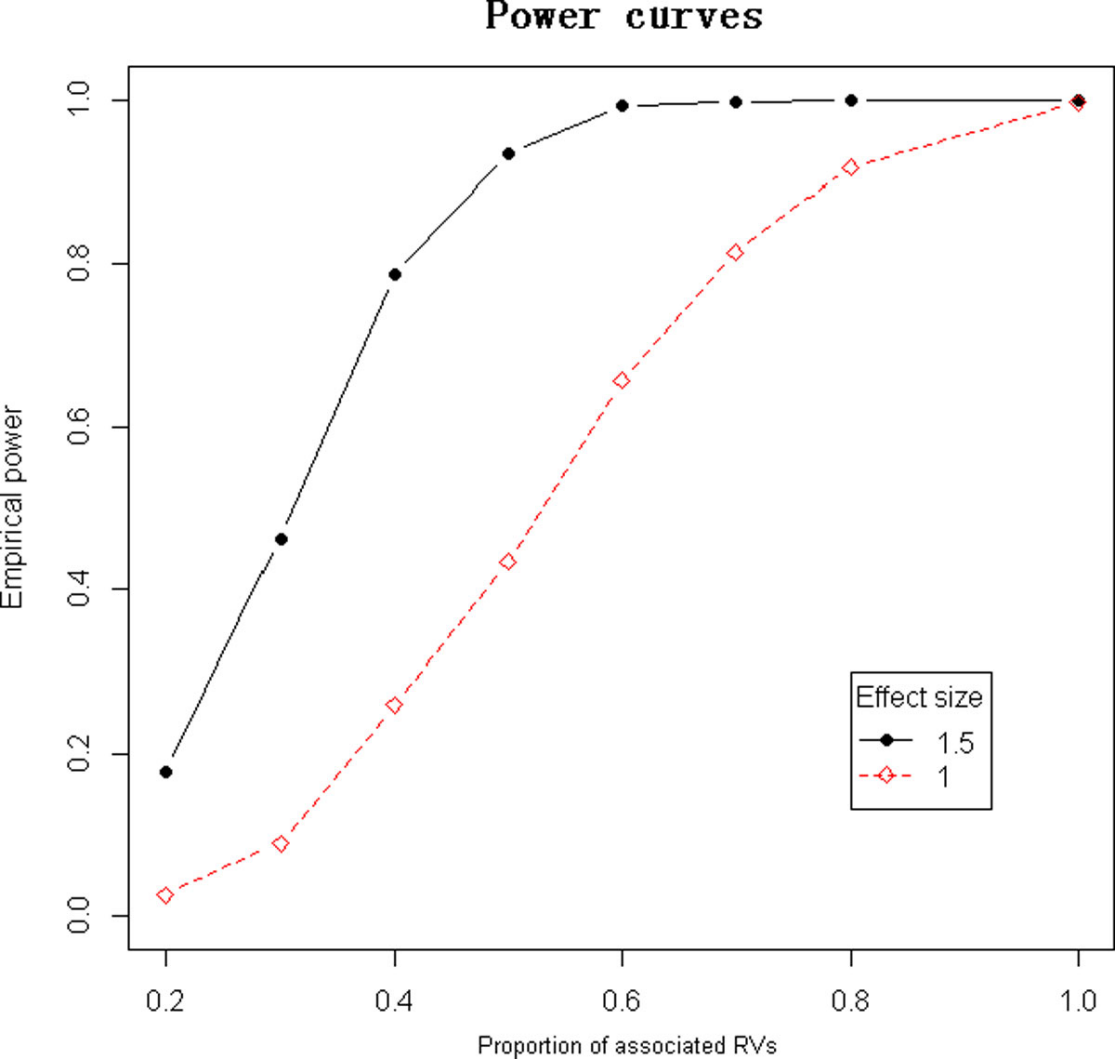
**Empirical power comparison between different vaules of $\beta_{1}$** . The red dashed line is the power curve for $\beta_{1}=1$. The black solid line is the power curve for $\beta_{1}=1.5$.

Unfortunately, for the Genetic Analysis Workshop (GAW)18 simulated data, only 275 trios can be incorporated in our analyses, owing to the large number of parents with missing genotype. Most causal SNPs with MAF less than 0.01 do not present minor alleles in these 275 trios, so they are not suitable data for testing our method. Consequently, we investigated the performance of FBCM by generating a variety of simulation scenarios involving different effect sizes and proportions of associated RVs. In particular, we consider the settings in which 20% to 100% of RVs are associated. The total number of families, the offspring in each family, and the total number of RVs are fixed at 300, 2, and 50, respectively. To generate genotypic data for each family, a proportion of the RVs are randomly selected to be causal, represented by an indicator vector *r*. Half the RVs are randomly selected to be neutral but are associated with the phenotype through population stratification, represented by an indicator vector *s*. The genotypic scores of the parents are independently sampled from Bern(2·MAF(k)) for the *k*^th ^RV, where MAF(k) is fixed as 0.005 throughout all RVs. Then the genotypes of children are obtained from parental haplotypes by random transmission, denoted by a 2 × 50 matrix ***G***. ***G ***is divided into the expected genotypic score matrix *E *and the deviation matrix *D *for the offspring in a family. The phenotypes of the 2 sibs in each family are generated from *N*(*β*_1 _• (***D *** × ***r***) + *β*_2_•(***E *** × ***s***), **Σ**), where *β*_1 _is the effect size, *β*_2_= 0.5 reflects the effect of population stratification, and $\text{Σ}=\left(\begin{array}{cc} 2 & 1\\ 1 & 2 \end{array}\right)$ is the covariance matrix to reflect the family structure. We set the hyperparameter $\sigma_{\beta }^{2}$ as 10^4 ^and tuned the BF cutoff equal to 2 so that the type I error rate is controlled below 0.005. Figure 1 shows the power curves with *β*_1 _= 1 and 1.5.

To evaluate FBCM using real data, the association analyses are performed by fitting our method to the data that use the full pedigree structure provided, with the entry for each variant being the estimated number of minor alleles carried. Our aim is to identify the genes and underlying RVs related to systolic blood pressure (SBP) throughout those 11 chromosomes among the GAW18 type 2 diabetes families. To better reflect the association between predisposition to hypertension and the variants, the highest SBP measured at the 4 examination points is selected as the phenotype for each individual. Log-transformation of the phenotype is performed to fix the skewness of the phenotype distribution. The age corresponding to the highest measured SBP is included in our model as a covariate to account for the significant correlation between age and SBP. Individuals with any missing data or without parental information are excluded, leaving 275 trios remaining.

**Figure 2:**
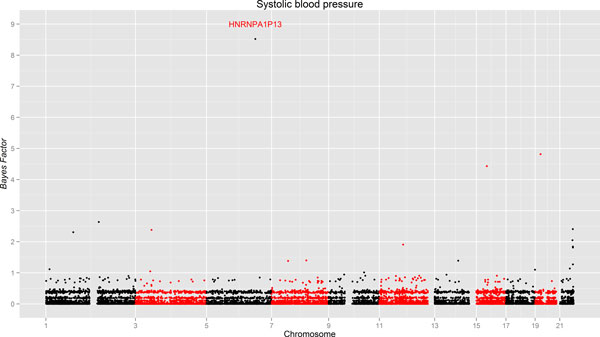
**Manhattan plot of BFs in the GAW18 study for the SBP**. The horizontal axis shows the chromosomal start positions of the genes. The vertical axis shows the BFs of the genes.

Using the gene information being obtained from Ensembl (http://www.ensembl.org/index.html), we investigated the genes on all 11 chromosomes. For each gene, only variants with MAF of less than 1% within the boundary of the gene are included in the analyses. The MAFs are estimated using 959 individuals in the dosage genotype data. The results are summarized using a Manhattan plot in Figure 2, which shows the BFs (see formula (2)) for the 16,759 genes across these 11 chromosomes. Table 1 presents the most significantly associated genes, with their BFs, estimated effect sizes, HUGO Gene Nomenclature Committee (HGNC) symbols (http://www.genenames.org/), and Ensembl gene IDs. The effect size conveys the estimated magnitude of the relationship between SBP and the transmission deviation. Our results show that most genes have BFs of far less than 1, and given only 275 trios in the analyses such results are not surprising. However, we still identify several genes with a BF larger than 2, which is the cut-off obtained from the simulation. The evidence of the association is substantial for those BFs between 3 and 10, based on Jeffery's grade of evidence [14], which is relatively subjective because BFs can be sensitive to many factors such as priors and number of RVs. More precise threshold values can be determined by permutation within or between chains in MCMC method. Although the potential influence of RVs on SBP is elusive, previous studies have identified a handful of genes with common variants (MAF>1%) associated with SBP [15]. Our results indicate that several genes with underlying RVs are potentially related to SBP and deserve to be further scrutinized.

**Table 1:** Most significant genes associated with SBP

Chr	Ensembl ID (ENSG00000-)	HGNC symbol	BF^1^	Effect size
5	213568	HNRNPA1P13	8.52	0.43
19	127529	OR7C2	4.82	−0.24
15	259500	RP11-138E16.2	4.43	−0.32

**Gene: HNRNPA1P13**				
Chr	Position	BF^2 ^(against null)	Index SNP	MAF
5	135764696	36.84	rs139658064	0.004
5	135765214	0.00368	NA	NA

^1 ^BF in formula (2).

^2 ^BF in formula (3).

Next, we investigated the underlying SNPs among the most related gene, HNRNPA1P13. The most significant SNPs based on their BFs in formula (3) are listed at the bottom of Table 1. The MAF information on these SNPs comes from the 1000 Genomes Project (http://www.1000genomes.org/). The larger BFs favor the evidence against the null hypothesis and indicate that positive deviation from the expected number of transmitted minor alleles drives the effect of the gene, while the BFs much lower than 1 suggest the effect of the deviation in the opposite direction. For example, given the effect size of gene HNRNPA1P13 is 0.43, SNP at position 135765214 with BF 0.00368 indicates that more transmitted copies of a minor allele from parents are likely to have a negative impact on the phenotype.

## Discussion

The FBCM proposed here is a novel statistical method for analyzing the association of RVs in pedigree data. The new methodology accounts for potential nonassociated variants by introducing random effects in a multiplicative way to approximate and capture the variant effects. The FBCM also takes into account situations in which some pooled variants can be associated with a phenotype through population stratification. Although the between-family component in our model can be integrated into the family-level random effect $\phi_{i}$, when the RV under investigation shows a significant between-family effect, our model performs better by capturing this effect to reduce the residual error. The model is based on the HQTDT, but expands the HQTDT by incorporating the collapsing information of the deviation from the expected genotypic score for a group of SNPs and at the same time maintaining orthogonality. Unlike the model selection method [9], our model employs random effects to predict variant-specific effects based on data and succeeds in boosting the sensitivity for gene association detection by collapsing the random effects of RVs. By taking advantage of the rare occurrence of minor alleles in an individual, the algorithm considers at most 2 sites in order to reduce the number of predictors, circumventing the huge computational burden involved in obtaining the full posterior distribution.

In variant-level analysis, our method improves the power to detect RV effects. The improvement of statistical power can be achieved by accounting for the random effects of all variants in a candidate gene through Gibbs sampling. Moreover, in the GAW18 data, it has been shown that the occurrence of a RV tends to be more common if a family member carries a minor allele. Thus, the family-based analysis is expected to have more power than the independent population-based analysis. The results show that our family-based method is able to identify both genes and individual SNPs significantly related to the phenotype, even in RV situations.

Our model can be further expanded in many ways. The appropriate link functions can be employed to handle other forms of phenotype, such as binary data. In this study, we focus on families without any missing data. However, for the trios with missing parental genotype information, the genetic score can be decomposed into between-family and within-family components by using only sibs genotypes. For the random effect distribution, the Bernoulli distribution is assigned as the prior distribution for the random effects of individual variants. For better modeling of the effects of individual variants, more sophisticated distributions can be employed.

## Conclusions

We have demonstrated that a novel FBCM can be applied to identify associations between RVs and quantitative traits for pedigree data. This method cannot only detect the gene effect, but can also pinpoint the underlying SNPs. Compared to other methods for handling RVs, our method based on family data improves statistical power by collapsing and accounting for all possible RV effects in a gene with population stratification controlled. Because the method allows for computational efficiency in obtaining the full posterior distribution, it is applicable to large-scale association tests. The results of our genome-wide analyses provide insights into the potential role of RVs in SBP.

## Competing interests

The authors declare that they have no competing interests.

## Authors' contributions

Both authors participated in designing the methods. He L, conducted the simulation, analyzed the data, interpreted the results and wrote the paper. Pitkaniemi co-designed the analysis and revised the paper. Both authors read and approved the final manuscript.
